# Practical Medicine

**Published:** 1877-02

**Authors:** 


### I. Practical Medicine.

1. *Athetosis.* Bernhardt. (*Clinic, Mo. Abstract,* August, 1876.

Dr. M. B., privat docent in Berlin, contributes an article to *Virchow's Archiv,* Band 67, Heft i., in which he attempts an analysis of those complex symptoms grouped by Hammond under the name athetosis (without fixed position). This disease is characterized by an inability to retain the fingers and toes in any position in which they may be placed and by their continued motion. These movements are not irregular, as in chorea, nor tremulous, as in paralysis agitans, but occur slowly, and with a certain degree of regularity. The will is almost powerless to control them, and they occur even during sleep. As a result of the continued activity of the muscles of the fingers, particularly of the thumb and index finger, a considerable hypertrophy of the muscular tissue of the forearm takes place. The sensibility of the skin is diminished; the prognosis, so far as regards a cure, is unfavorable, all therapeutic means having thus far proved futile. Up to the present time no *post-mortem* examinations have been made. Hammond suspects a lesion (sclerosis) of the corpus striatum or of the thalamus opticus. His first two cases were those of two men, one of whom, a very intemperate person, had suffered attacks of epilepsy and delirium tremens; the other was of a tubercular tendency, and his father and grandfather had been excessive drinkers. After several decided apoplectic attacks, he became aphasic, and, in addition, exhibited the usual symptoms of "athetosis." Up to the year 1873, Hammond had encountered six cases of this affection, two of these being women; three were drunkards. In the *Medical Times and Gazette,* 1872, Vol. I., Clifford Allbut described the case of a woman, aged fifty-five, presenting these symptoms, but in whose case no suspicion of tippling could be entertained. The report, however, was accompanied by an expression of doubt on his part of the correctness of classifying it under the same head as Hammond's disease. It differed from the latter chiefly in that the movements ceased entirely during sleep. Currie Ritchie, in the same journal (page 342), and with the same reservation, reports the case of an engineer, aged fifty-nine, non-syphilitic, and not given to drink, who, after an apoplectic seizure, remained speechless (aphasic?) for three months, at the end of which time he presented these continuous uncontrollable movements of the right fingers and toes.

In the *Journal of Mental Science,* Vol. XIX., 1874, Gairdner relates the case of a boy, not predisposed to nervous maladies, who manifested the same symptoms; absolute rest and strict attention served to diminish the number and force of the movements, but they never ceased entirely except during sleep.

These are all the cases thus far carefully described. It will be observed that the only minute accounts have been rendered by Americans and English, as the case mentioned by Eulenberg, even in his own estimation, scarcely falls under this head.

The writer then gave the following report of a case observed by himself:

Robert Krüger is the twelve-year-old son of his still living and healthy father. The mother, likewise in perfect health, has borne twelve children, of whom seven still live. Four died in early infancy; the fifth, a son, was in good health until sixteen years old, when, having the toothache, he went to a dentist, who broke off the diseased tooth. From this time began a "restlessness" of all the muscles of his body, which led his parents to take him to the Berlin poliklinik. The symptoms improved after a few applications of the constant current, but increased very much after the removal of the root of the tooth, and he soon afterwards died.

Of the children still living, a daughter, ten years of age, had violent choreic symptoms for over six months. She has now recovered, and is strong and healthy, but with every variation of the weather manifests sudden violent changes of disposition, and has severe jerking motions of one side. The five remaining children are healthy and active.

This boy Robert was also, up to his fourth year, in good health. After an acute illness, lasting for several weeks (from all accounts this seems to have been exanthematic), irregular motions of the right foot and hand were observed. Although these continued, he went to school at the usual age, and progressed with his studies like other children, but was often observed to cry. He learned to write with his left hand.

The patient is a boy quite well developed for his age; has no fever; attends school; appetite and sleep are good; he has frequent headaches, particularly in the neighborhood of the forehead and temples, which parts, especially the left side, are sensitive to pressure. His cerebral functions seem perfectly intact. Nothing abnormal can be discovered in the reaction of the pupils nor the movements of the eyes. The tongue, on being protruded, deviates very slightly to the right, and exhibits involuntary contractions. Both tonsils were enlarged. In state of quietude, no difference of the two sides of the face could be observed; but when the patient attempts to shut his eyes, the motion is much more rapid on the left side, and the left angle of the mouth is somewhat higher than the right. During the act of laughing the mouth is drawn a little more toward the right. Now and then, perhaps twice within a quarter of an hour, and when speaking, he draws the mouth a little to the right. His voice is low, but clear and distinct.

Motility and sensibility of the entire left half of the body are perfectly intact. Even on the right the motions at the shoulder and elbow are readily made, but more slowly and weakly than on the left side. No involuntary movements are ever observed in the head, shoulder or forearm of the right side during sleep or in a state of excitement. The right lower extremities, the thigh and leg, are perfectly quiet. The foot and toes, like the hand and fingers, seem to be the only parts involved. Now and then he suffers pain of a shooting character in the entire right upper extremity.

The patient walks alone; now and then his foot bends laterally, while his toes often turn under. He can stand for a considerable time on his right foot. If he be seated quietly, and his attention wholly occupied with something else, the right hand and fingers will remain in perfect rest. So soon, however, as his attention is attracted to these parts, a remarkable restless play of the fingers begins. In tolerably rapid succession these are ab- and adducted, flexed and extended; the thumb, the index, and the little finger are the ones affected, whilst the two others remain quiet. The joints of the hand seem meanwhile to be immobile, while a continuous muscular play is observed on the flexor and extensor surfaces of the right forearm; the configurations of the muscles and tendons of the forearm are undergoing continual changes, which are quite noticeable under the skin. The sensibility of the entire right side of the body, face, trunk, and extremities, as compared with the left, is decidedly diminished. Now and then a prolonged effort of the will succeeds in controlling the movements, but just as often they become much more violent. The right forearm is more voluminous than the left. The exact amount of increase due to these movements cannot be determined, as the left is also larger than it would otherwise be, since the boy has been using it in lifting, writing, eating, etc., ever since the fingers of the right side began their involuntary play. The hip and knee joints are at rest. There is a slight lateral displacement of the foot and a distinct plantar flexion of the toes. Sometimes, particularly at night (in which they differ from the fingers), decided involuntary movements of the toes take place. So, at least, say his relatives; his father has observed these motions during his son's sleep.

The continual motion of the muscles of the forearm rendered an electrical examination of but little value; but it was shown that the muscles supplied by the ulnar and median nerves responded promptly to the faradic and constant currents.

The heart-sounds, examined repeatedly, are loud and clear.

The malady has now remained unchanged for seven years; galvanization, attempted for months (by the passage of a weak constant current through the head), the regulation of diet, and the administration of tonics, have been followed by no results worthy of mention.

There can be but little doubt that the history of this case corresponds essentially with that series of symptoms denominated by Hammond "athe-osis." As in other cases, we find here the restless play, especially of the fingers and less of the toes, during perfect quietude of all other parts of the extremities; we find the peculiar position of the foot and toes, the anæsthesia of that half of the body, the diminution in strength, even though in but slight degree as compared with the healthy side, the hypertrophy of the muscles kept in such continual motion; we observe, also, the long continuance of the malady and the seeming impossibility of cure.

The cases thus far observed, of which we have reliable knowledge, affected three men, one woman, and one boy; both sexes, and early and late life. Of these, Allbut's woman and Gairdner's boy had no hereditary predisposition, and no indications of previous disease of the central nervous system. On the other hand, one of Hammond's was a drunkard and epileptic; and the second, the son of a toper, became aphasic after an apoplectic attack. This was also the history of the three male cases reported by Ritchie. The history of the boy Krüger demonstrates a predisposition in the family to nervous diseases, particularly to chorea. That he acquired this affection after a febrile exanthema proves nothing; but it is somewhat remarkable that in his case the nervous disease developed into something so resembling chorea, a disease with which at first sight it would be most likely to be confounded. Now, then, we come to the point of greatest importance, namely, whether in the collection of symptoms described by Hammond we really have a new disease, one not heretofore described. It might, perhaps, be mistaken for that other nervous malady, *paralysis agitans.*

The most essential points in the symptomatology of this disease are a tremulousness of the voluntary muscles and their gradual wasting away. It is a disease of advanced age, although a few writers (Meschede, Huchard) have observed cases in children; at all events, these early cases belong to the greatest of rarities. The absence of any great degree of paralysis, in these cases of athetosis, which their long continuance might have led us to suspect, the absence of all tremors of the head and remaining parts of the upper extremities, the absolute limitation of the involuntary motions to the most peripheral portions of the extremities, the restless movements of the fingers (which cannot however be classed as "tremulous," as it represents more correctly a motiveless grasping), all seem to indicate the incorrectness of identifying these two sets of symptoms. The picture of the affection just given likewise contraindicates its classification with insular degeneration of the cord, another peculiar affection heretofore classed with the great group of chronic diseases of the spinal cord. In this disease uncontrollable tremulous motions are also observed, but these never occur so long as the patient undertakes no voluntary motion; in fact, the point recognized by all writers as distinguishing this from paralysis agitans, is that these tremors occur only when the patient is attempting a voluntary motion. In that disease, again, the motions are tremulous, which, as before stated, is not the case in athetosis; further, in insular sclerosis they are not confined to the fingers and toes, nor are disturbances of sensibility observed in the majority of cases. Finally, in sclerosis the origins of many cranial nerves are affected by the chronic degenerative process, and so complicate the symptomatology by affections of the ocular muscles, of those of the tongue, the larynx and the pharynx, that these complications become the almost characteristic symptoms of the disease; this is not the case with athetosis, thus affording a proof that the latter is a separate and distinct disease, and should not be mistaken for or classed with sclerosis. Still further, it must be remembered that sclerosis, like paralysis agitans, has rarely, if ever, been encountered in childhood. We come back finally to the point from which we started; an impartial observer of a case of athetosis would probably first suspect that he had to deal with hemi-chorea, or unilateral chorea. [The writer lays special stress upon the word " unilateral," as all the cases thus far have been right-sided.]

The patient whose history is above narrated has had for years an affection coming on after a long-continued illness; Hammond's first case (an epileptic) was in an unconscious condition (delirium) for six weeks, at the end of which time the right upper extremity began to manifest the motions characteristic of athetosis; in his second patient they came on after an apoplectic seizure followed by aphasia; in Ritchie's case, too, these followed an apoplectic attack succeeded by a three months' aphasic condition. Here, too, the right extremities were the affected ones. These considerations lead Bernhardt to place athetosis in that group of affections which have lately been designated under the names Post-paralytic chorea (Weir Mitchell, *Amer. Journ. Med. Sciences*, 1874, page 352,) or Hemichorée post-hémiplégique (Charcot, *Progrès Médical*, 1875, Nos. 5 and 6). According to these authors, a choreic condition is frequently developed in the paralyzed limbs after a long continuance of hemiplegia. There is generally also an anæsthesia of the skin and of the organs of sensation on the paralyzed side. Charcot locates the affection on the opposite side of the brain, at the posterior portion of the thalamus opticus, the nucl. cand., and the posterior portion of the foot of the corona radiata.

The writer agrees with Hammond and Charcot in considering this the location of the lesion in athetosis. Still we have as yet no positive proof of this, as no *post-mortem* examinations have yet been made. The previous apoplectic attacks in several of the patients, the aphasic condition, and the disturbances of sensation in the affected parts afford pretty conclusive evidence.

He does not, therefore, feel inclined to admit the correctness of Eulenberg's view which locates the lesion in circumscribed portions of the cortical substance. Affections of this character have never been observed to last for years, nor have circumscribed groups of muscles exhibited the movements peculiar to athetosis; nor have the motions been continuous, quiet and regular, but have recurred as separate attacks at considerable intervals; there have been jerking movements in the extremities which ceased after a time, to be succeeded by paresis. These cases do not last so long as those of athetosis, nor do they follow that fixed course we have observed in the ones thus far examined.

It was this peculiar phenomenon of the limitation of the involuntary, choreic movements to the fingers and toes which induced Hammond to adopt the new name "athetosis." The symptoms in general resemble those of hemi-chorea, but differ in some very important particulars, for in the latter affection the motions are not confined to the extremities, but extend also to the face and the organs of speech and deglutition; besides, hemi-chorea affects by preference the left side. Although he believes that in this affection we have nothing more than a modified chorea, he would, for the present at least, retain the term athetosis, as the affection differs from all others in some important features, and as the term itself is based only on symptoms, it should be allowed to remain until sufficient anatomico-pathological observations have been made to establish a name for all time.

2. *Lesion of the Cerebellum and Diabetes Mellitus.* MOSLER. (*Berl. Klin. Wochenschr.*, *N. Y. Med. Record*.)

In a fatal case of diabetes mellitus, which was treated in Mosler's clinic, and in which there had been no disturbances of sensation, or motion, or of the special senses during life, the autopsy revealed a well marked spot of inflammatory softening about the size of a pigeon's egg, in the nucleus dentatus of the left hemisphere of the cerebellum. The theory of the neuropathic origin of diabetes rests on sound practical grounds, and there can be little reason for doubting that in this case the increased secretion of sugar was excited by the central affection, especially as Eckhard has shown that injury of the second lobe of the vermis cerebelli in rabbits will artificially excite diabetes. It is strange that such a well marked lesion was not attended by any signs of disease of the central nervous system during life.

3. *Functional Spasm.* MITCHELL. (*Am. Jr. Med. Sc.*, Oct., 1876.

Dr. M. describes a number of cases differing in several particulars from writer's cramp and the spastic disorders of shoemakers, tailors, etc. Such cases are either those wherein, (1) natural muscular actions are exaggerated, with sometimes spasmodic activity in remoter groups, or (2) action of one group of muscles causes spasmodic acts, tonic or clonic, in remote groups of muscles not involved in the original movement, or (3) standing or walking occasions general and disorderly motions affecting the limbs, trunk and face, and giving rise to general spasm without loss of consciousness.

One case was that of a maker of watches, whose work required him to be constantly picking up tiny screws; he had followed this employment thirty years. His general health was perfect, but for two years frequently on picking up a screw his thumb and forefinger would become locked fast with a powerful grip, which would last from ten to thirty minutes. Often the spasm would cause the fingers to be cut by the screw. The spasm occurred ten or twelve times a day. The spasms occurred oftenest when his body was in the usual position for work. There was no pain, and only at times slight tremor. He had all sorts of treatment. Nothing but rest did him any good.

Another patient was a sawyer of wood, of forty, temperate and healthy. He had occasionally a spasm of the biceps, which would occur just as he had the saw drawn back for a downward motion. The spasms were violent, the forearm being drawn tightly up against the arm and with such power that a force of eighty pounds pulling against the forearm only caused the spasm to slowly give way at the end of five minutes. The spasms sometimes lasted for several hours. An attack occurred usually once or twice daily. The spasms ceased after several months under the use of the induced current—one pole on the outside of the biceps, below, and one on the belly, above, and the strongest endurable current being passed for two hours daily—and hypodermic injections of atropia in the belly of the muscle.

An officer in rugged health was shot through the forearm and arm at the same moment, as he was in the act of drawing his sword. His hand grasped the sword hilt with such a spasmodic grip that the left hand was required to pry apart the fingers.

Both the median and musculo-spiral nerves were divided and he had for many months tormenting causalgia (burning pain) in the palm. He eventually recovered from this. After the war he had frequent attacks of violent spasmodic closure of the hand, which would come on when he was holding something in the hand, and always with a sharp pain at the point of division of the median in the palm. The spasm would last two to ten minutes, and the palm was often cut by the violence with which the nails were driven into it. The pain subsided as the spasm came on, and the latter was painless.

Twelve successive blisters of the palm were followed by a disappearance of the malady, and at the end of a year it had not returned.

A plumber forty-five years old was struck on the back of the head and knocked down, in 1862. He had stiffness in the muscles at the back of the neck, and in three months his head was slowly drawn more and more to the left. Afterward he had frequently horrible spasms in the trapezius, sterno-cleido-mastoid, tracheloid and spinatus muscles. "Every minute, at least, the head was jerked backward and twisted to left, the shoulders drawn up, and a storm of rapid but energetic spasms swept over the facial muscles." He improved greatly while taking gelseminum and bromides in liberal doses.

Soon afterward he began to have spasms of the submental muscles. While eating his mouth would suddenly be jerked open and remain so until he would close it with his hands and chew a little more, when it would return. "When ready to swallow his face presented a picture of terror. He would suddenly muster courage and swallow the contents of his mouth at a gulp. Then instantly the jaw flew open, the head was drawn back and down on the left shoulder, the face was convulsed, sweat broke from his forehead, and the attack was at an end."

One case was of spasmodic "exaggerations of walking." The patient was a boy of six, and the trouble came on slowly after an attack of measles. The left leg acted normally. "As he swung the right forward and raised the toes to avoid touching the ground, there was at first a sudden spasm of the tibiæ and peroneal muscles, so that the tip of the foot was jerked up too high." This suddenly relaxed, and as the body was raised on the ball of the foot it was lifted abruptly by a spasm of the gastrocnemii, which continuing jerked the leg backward—a true string halt. While at rest on his back he moved the leg without spasm.

In another case—a child of seven—there were symptoms similar to the above, but some that were different. She did not learn to walk until her fifth year. In flexing the foot and the muscles above the knee there was a rigidness, flexion being resisted by the antagonizing muscles so that the motions were made slowly and with difficulty—a condition he describes as "lead pipe leg." "Every time the foot came down the gastrocnemius muscle contracted violently," and lifted the child with a jerk. The tendons of the calf were cut on both feet, with the result of marked improvement. Intellectually this child was normal.

The next case was a sallow lad of seventeen, who had measles at the age of nine, recovering slowly with weakness of the legs. As his legs had grown strong a stiffness had come over him embarrasing his movements. The legs were developed to nearly an equal degree. While recumbent they were quiet; standing, the back was drawn into a bow, and a strong effort of the will was needed to keep the left leg quiet—spite of effort it would occasionally be jerked upward. Walking, both gastrocnemii jerked, making a string halt. When seated he kept the leg still by crossing it over its fellow. Electricity in all forms failed of any good in this case. A long series of atropia injections into the calf did a little good. Repeated cross sections of the back muscles were made at different levels, and slight benefit ensued. Finally an apparatus limiting the extent of motion was applied.

A watchcase-maker, thirty-three years old, had a bad family history. A sister, two aunts and his mother had palsies in middle life; an uncle had epilepsy and a cousin dementia. He was always nervous, never had syphilis and was well until 1865, when for two days he was unconscious from sunstroke. Ever since he had been made weak by summer heat. In 1874 he first noticed weakness of the legs in walking; he was obliged to stop as if to gain power; after which the right leg would drag for a time. Pain now occurred in the back of the neck and lumbar spine. In January, 1875, tremors began in the left arm, which increased until in three months a strange state existed. In sleep there was no movement. Soon after waking the left hand began to twitch, the fingers moving as if playing a violin. "The slightest movement of any other limb, speaking or eating" caused the arm to execute a constant motion of striking the bed or his side, the limb being the while extended. On walking this motion was exaggerated and resembled the rapid movements of a pendulum. The rate of motion was tolerably uniform, and was from 157 to 200 to the minute. It was as rhythmic as the heart beat. If the right hand was extended at a right angle with the body the rate would suddenly increase to 200, to fall back to 160 on bringing the arm at rest by the side.

"When there was no pendulum spasm he could perform with the left arm any voluntary act not involving the hand, which never ceased to twitch; but while the swinging spasms lasted he could execute no volitional act," and the effort to move the limb increased the spasms greatly.

"Excitement and emotion, and all forms of electricity added to the force of the motions, but voluntary movement of other limbs increased the number more than the force." Attempts at passive motions caused pain in the occiput.

"He has power to stop the spasm by certain manœuvres. If he seizes the left hand with the right and flexing the left arm, holds it there in a kind of general spasm, the left hand for a moment seems to struggle with increased violence; he totters; the face is convulsed; there is horrible pain in the back of the head. Then he gently releases the left arm, which, save for a slight tremor or twitching of the unquiet fingers, remains at rest, and may not move in violent spasm for an hour or more, and is sometimes nearly still for twelve hours."

There was no loss of consciousness, anæsthesia, ocular trouble or aural defect. No treatment had been of any service.

Dr. M. closes his paper by a record of three cases in which, while the patient was at rest, prone, or as in one case seated, no spasms existed; but when the upright posture was assumed there was general convulsive motions, sometimes persisting, sometimes not.

4. *Genital Irritation as a Cause of Nervous Disease, etc.* HAMILTON. (*Am. Jr. Med. Sc.*, Oct., 1876.)

"Continual morbid peripheral irritation sent from the genitals, either to the bladder, cord or medulla oblongata," may cause any of the following symptoms; paresis, including paraplegia; paresis of the muscular fibres of the bladder; disturbed sensation—hyperæsthesia, anæsthesia or dysæsthesia; priapism, local hyperæmia; choreic movements, transitory contractions; loss of consciousness, impaired memory, irratibility of temper, melancholia and dementia. Many or few of these symptoms may co-exist. In children congenital defects and bad habits, and in adults venereal excess, and more rarely stricture and urethral difficulty, seem able to cause them. Sayre has reported ten cases of children who suffered with some of these symptoms. Six of the patients were between three and four, two were two and two were five years old. Eight were boys and two were girls. "In the boys there was generally congenital phymosis with agglutination of the prepuce and glands; in the girls an elongated and inflamed clitoris. Symptoms of want of power or in perfect locomotion, adduction of the lower limbs, atrophy of the muscles of the thighs and legs, incontinence of urine, and various mental disturbances of greater or less gravity, such as hysterical laughter, irritability of temper, or imbecility, were also presented." Operation was followed by recovery in from a few hours to as many weeks.

Dr. F. H. Brown, of Boston, had a case of paraplegia in a boy of ten. There was incontinence of urine. A severe balanitis with preputial constriction existed. Recovery was rapid after circumcision.

Dr. H. relates a cases of a boy of seven, well nourished and rosy. There were irregular convulsions in the lower extremeties when recumbent. There were choreic movements of the arms. When held up he was unable to stand, not from paresis but from lack of power of co-ordination; the legs became rigid and the toes adducted. He was unable to speak, but made "queer sounds," and he had choreic movements of the face. There was no imbecility and he seemed fearful of being hurt. His functions of nutrition were perfect, and he slept well.

He had congenital phymosis, the prepuce being firmly attached to the glans; the preputial orifice was small and surrounded by a rigid ring of toughened skin. Titillation did not cause immediate erection, nor increase of spasmodic movements. There was no sensitiveness to pressure along the spine.

Another case was that of a pale delicate girl of five who had never been able to stand. Intelligence was unaffected, no atrophy of the legs existed, nor was there lack of sensation and proper temperature. On being held upright the legs became stiff and the toes crossed each other, and one foot seemed inclined to cover its fellow. The heels were drawn up from contraction of the sural muscles. On lying upon her back the thighs were always flexed upon the pelvis, and in this position she slept. When sleeping, too, the head was drawn back by the trapezius and other muscles. The clitoris was large, cyanotic and erect. There was no history of worms.

The third case was a boy of seven. From early infancy he had had convulsion at varying intervals. They were short, the pale stage was absent and they were not followed by sleep. The bodily condition was good. A phymosis and contracted preputial orifice existed. There was no incontinence of urine.

The fourth case was a well developed boy of nine, of nervous temperament. The birth of this child was by a protracted labor and use of forceps. When two or three days old he had convulsions. He only began to talk at three years, and then with much difficulty. A year later he began for the first time to stand alone and to walk. There was no history of painful erections, but there was nocturnal incontinence. The whole body manifested choreic movements, as in the first and second cases above quoted. This was most manifested on his attempts at standing. He had phymosis, the preputial orifice only admitting a small probe.

Dr. H. infers that in these cases and cases like them, the convulsive movements, especially of the lower extremities, the spastic contractions of the flexors of the thighs and legs are connected with some irritation of the glans or clitoris; that usually in children beyond the irritability there are no mental symptoms; that exaggerated power is the rule and atrophy the exception; and that inco-ordination is common.

5. *Metrical Weights in Prescriptions.* MAISH. (*The Med. and Surg. Reporter*, Sep. 9, 1876.)

M. thinks that the difficulty of introducing this most useful and desirable system of weights in our prescription writing is due chiefly to the alteration of values which it would compel us to learn. The identity of the system with our decimal monetary system will, he thinks, make it naturally convenient.

The metrical system is now used in medicine and pharmacy as well as as in the physical sciences in nearly all civilized nations except those speaking the English tongue.

The pharmacopœias of Continental Europe express all quantities by weight, be the material liquid or solid. This is by far the best way, and once learned is most accurate and convenient. "The difficulty of writing prescriptions by weight instead of measure" has been overestimated.

Of the officinal liquid preparations prescribed for internal use, the alkaline solutions, the dilute acids and solutions of some of the salts (ammon. acet., potass. cit.) about equal in bulk an equal weight of distilled water. Tinctures and fluid extracts differ more from water and among themselves. Ether (sp. grav. .750) has a volume 1⅓ time its weight of water—three parts by weight of ether occupy the space of four parts of water. The relations of weight to volume of spts. eth. nit. (sp. gr. .837) and spts. eth. comp. (sp. gr. .815) is nearly as 4:5; of glycerine (sp. gr. 1.25) as 5:4; of syrups nearly as 4:3; of chloroform (sp. gr. 1.48) nearly as 3:2.

Remembering the relative density of officinal liquids, prescribing by metrical weights is easy. For all practical purposes one gramme is equal to 15 grs., one fluid ounce of water equals (within eight grs.) 30 grammes. 15 grammes of water, or its equivalent in other fluids, is to be taken as equal to a tablespoonful; 7.5 to 8 grammes to a desertspoonful and 3.7 to 4 grms. to a teaspoonful.

Applying these values to the heavier and lighter liquids it will be seen that

| | |
|---|---|
| 15 × ¾ = 11.25 grammes ether, | 15 × 4.5 = 12 grms. spts. ether, nit. |
| 15 × 5-4 = 18.75 grms. glycerine, | (or comp.) |
| 15 × 3-2 = 22.50 grms. chloroform, | 15 × 4-3 = 20 grms. syrup, |

Are in measure equal to about half a fluid ounce, and that the deviation from this measure is in each case considerably less than the difference in the amounts obtained by scant and full measurement with the same teaspoon, or between different patterns of that very useful domestic utensil.

The average dose of these liquids, expressed in metrical weights, are therefore:

| | | |
|---|---|---|
| Ether, | 11.25:8 = 1.40 grms. | or ½ teaspoonful. |
| Spts. Eth. c. or n. | 12.00:4 = 3.00 grms. | or 1 teaspoonful. |
| Chloroform, | 22.50:8 = 2.80 grms. | or ½ teaspoonful. |
| Glycerine, | 18.75:4 = 4.70 grms. | or 1 teaspoonful. |
| Syrups (some), | 20 00:4 = 5.00 grms. | or 1 teaspoonful. |

For conversion of grain weights of solids into grammes, close enough approximations are made by dividing the number of grains by 15.

The smallness of the error is seen by the following:

| | | | | |
|---|---|---|---|---|
| grs. lx | give 60-15 = 4.00, | absolutely 3.887, | difference | 1¾ grs. |
| ℥ j | give 480-15 = 32.00, | absolutely 31.100, | difference | 13 9-10 grs. |
| gr. x | give 10-15 = 0.66, | absolutely 0.648, | difference less than | 1-5 gr. |
| gr. viii | give 8-15 = 0.53, | absolutely 0.518, | difference less than | 1-5 gr. |
| gr. iij | give 3-15 = 0.20, | absolutely 0.194, | difference less than | 1-10 gr. |
| gr. ij | give 2-15 = 0.13, | absolutely 0.129, | difference nearly | 1-65 gr. |
| gr. j | give 1-15 = 0.066, | absolutely 0.065, | difference nearly | 1.65 gr. |

In prescribing, the quantities should be expressed in grammes and decimal fractions thereof, no signs or abbreviations being necessary.

Following are sample prescriptions written in the usual manner with their metrical equivalants attached. The author thinks it must be apparent there is less liability to error by using the latter method.

| | | | |
|---|---|---|---|
| ℞ | Pot. iodidi | ʒ ij | 8.00 |
| | Iodini | gr. ij | 0.13 |
| | Aquae | f℥ ss | 15.00 |
| | Syr. sars. c. | f℥ iiiss | 140.00 |

Dose, one tablespoonful, containing one gramme (15 grs.) pot. iod. and 0.016 (¼ gr.) iodine.

| | | | |
|---|---|---|---|
| ℞ | Potass. Nitrat. | ʒ iss | 6.00 |
| | Vini Antim | m. xl | 2.66 |
| | Tr. digitalis | fʒ i* | 3.50 |
| | Mucil. acaciæ | fl ℥ ss | 20.00 |
| | Aquæ | f℥ iij | 90.00 |
| | Syr. Aurantii | f℥ j | 40.00 |

A tablespoonful of this mixture contains 0.66 (10 grs.) potass. nitrate, 0.0012 (1-54 gr.) tartar emetic and 0.055 (5-6 gr.) digitalis.

| | | | |
|---|---|---|---|
| ℞ | Morph. sul | gr. i | 0.066 |
| | Pulv. digitalis | gr. vi | 0.40 |
| | Sacch. alb. | ℈ iss | 2.00 |

M. Div. cht. No. xii.

Each powder contains 0.0055 (1-12 gr.) morph. and 0.033 (½gr.) digital.

| | | | |
|---|---|---|---|
| ℞ | Quin. Sul. | gr. xii | 0.80 |
| | Pulv. opii | gr. iii | 0.20 |
| | Syrupi | q. s. | |

Fiant. pil. No. xii.

Each pill contains 0.066 (1 gr.) quinia and 0.016 (¼ gr.) opium.

| | | | |
|---|---|---|---|
| ℞ | Atropiæ | gr. ss | 0.033 |
| | Alcohol | q. s. | |
| | Adipis | ʒ j | 4.00 |

M.

Atropia ointment.

6. *Multilocular Cerebro-spinal Sclerosis.* GERHARD. (*Phila. Med. Times*, Nov. 11, 1876.)

In remarks upon this disorder G. says the lesion consists in the developement of sclerotic patches or islands in the substance of the brain and cord. The islands are irregular in form, and in the cord are scattered, generally, throughout its whole length and without respect to its division into columns and gray matter. Some preference is shown, however, for the antero-lateral columns. In the brain the gray matter of the convolutions usually escapes; the most frequent parts effected are the walls of the lateral ventricles, the septum lucidum, the pons and floor of the fourth ventricle.

The morbid change is a localized hyperplasy of the normal connective tissue, and, through pressure and absorption, a substitution for the nervous tissue proper.

The most marked symptoms are increasing loss of power in the extremities and a peculiar tremor occurring only with voluntary movement. There are also vertigo, frequently a peculiar difficulty of articulation, some disturbance of vision, nystagmus, and a characteristic facies. The tremor

* Representing 7½ grains digitalis; menstruum diluted alcohol. (This tincture to water is as 7:8.)

is peculiar. It is usually preceded for some time by the weakness, but not always so. It does not exist during repose of the muscles. It is large and rhythmical like the movements of chorea.

The trembling of paralysis agitans is unlike that of scattered sclerosis in being constant, and lessened instead of increased by voluntary effort. In sclerosis of the posterior columns or progressive locomoter ataxia, while the movements are irregular as is the disease being considered, they are always exaggerated and more wild on closure of the eyes; in insular sclerosis a patient with his eyes closed, being told to put his finger on a given spot on the face is perfectly able to do it notwithstanding the violent tremor.

The paralytic symptoms develop slowly; they begin usually in the lower extremities, one alone it may be. The paralysis of the lower extremities may in years become complete; in the upper limbs it seldom does, and the sphincters are rarely involved. There is seldom any loss of sensibility. Treatment has been almost useless. Corrosive sublimate in small doses long continued has given transitory benefit, so has nitrate of silver, and increasing doses of hyoscyamus have lessened the tremors.

Four cases are reported. In the first—a man of fifty-seven—the loss of power in the legs began seven years before. A year later the arms began to fail in strength, and then was there first noticed tremors or movements in all the extremities. Later his speech was effected, and in three years he was unable to work. The patient walked with a cane, moved slowly and with difficulty could raise the feet from the floor. There was no muscular wasting, or loss of sensibility or electrical response. The mind was impaired, the speech thick and deliberate, the eyesight poor and beginning atrophy of the fundus.

In case two there was the same order of occurrence of the weakness but the tremor began in the upper extremities. There was giddiness and the face was drawn to one side. There was no speech difficulty. The weakness and tremors were the cardinal symptoms.

Case three differed from the preceding in that the muscular trembling was noticed before the weakness. The gait was shuffling, and there was a tendency to festination. There was occasional vertigo and the eyes were normal.

In case four there were the typical symptoms, and in addition a condition of half idiocy. There was a subjective feeling of numbness, but the æsthesiometer proved the sensibility to be quite normal.

7. *The Cure of Diphtheria.* Cialtaglia, of Rome. (*Lancet*, Oct., 1876.)

Dr. C. reports eight cases to illustrate his flattering success with the use of large doses of chlorate of potash internally and hydrate of chloral locally in this affliction. All these cases recovered. The chlorate of potash is administered in solution of 20 grammes to 140 grammes of water —a tablespoonful being given every one to three hours, according to the age and severity of the case.

The chloral is dissolved in glycerine (four to twenty grammes respectively) and the diphthertic spots are touched with the mixture three or four time daily.

"The moment the chloral and glycerine was applied the false membranes ceased to spread, while the characteristic fetor from the mouth entirely disappeared on the first, or, at latest, the second day of application."

8. *Nestlé's Food for Babies.* PUTNAM. (*The Boston Med. and Surg. Jour.*, Nov. 9, 1876.)

Nestlé's Lacteous Farina, made in Vevey, Switz., is claimed to have some advantages over the other numerous preparations of food that are in use here, namely: Its dry form and the mode of preparation for use—water only being used. The food consists almost entirely of milk in the form of powder, and is mixed with bread baked for the purpose from the best flour. The crust being the most nutritious part is alone used. The milk is obtained fresh from the dairies; after having been tested it is received into steam heated vessels, and condensed in a partial vacuum at a nearly uniform temperature, not above 120° F. The product of this is a fine powder of milk and bread crust.

Lebert, on examination, found grains 1-12500 of an inch in diameter, and fragmentary parts of starch. The food is easily prepared for use, one part is mixed with from six to ten parts of cold water, which is then boiled while stirring. Any unskilled hand may prepare it.

Ehrendorfer, of Vienna, has reported 20 poorly nourished and 40 sick children averaging from five to twenty months old. Fifty-one of these continued to take the food until they were well, while the balance of them discontinued its use, either because they disliked it or because they failed to improve.

Ehrendorfer claims that the food is especially valuable in all cases where the mother's milk is not sufficient in quantity, and in diarrhœal diseases caused by weaning. The author's experience with the food is essentially the same as that of Ehrendorfer, and he concludes by saying that "generally it was well liked and well borne, occasionally it was not retained by the stomach or was not liked by the baby." W. F. L.

